# A streamlined method for the design and cloning of shRNAs into an optimized Dox-inducible lentiviral vector

**DOI:** 10.1186/s12896-017-0341-x

**Published:** 2017-02-28

**Authors:** Sander B. Frank, Veronique V. Schulz, Cindy K. Miranti

**Affiliations:** 1 Laboratory of Integrin Signaling and Tumorigenesis, Van Andel Research Institute, Grand Rapids, MI USA; 2 Genetics Program, Michigan State University, East Lansing, MI USA; 3 Department of Cellular and Molecular Medicine, University of Arizona Cancer Center, 1515 N. Campbell Ave, Tucson, AZ 85724 USA

**Keywords:** pLKO, shRNA, Lentivirus, Inducible

## Abstract

**Background:**

Short hairpin RNA (shRNA) is an established and effective tool for stable knock down of gene expression. Lentiviral vectors can be used to deliver shRNAs, thereby providing the ability to infect most mammalian cell types with high efficiency, regardless of proliferation state. Furthermore, the use of inducible promoters to drive shRNA expression allows for more thorough investigations into the specific timing of gene function in a variety of cellular processes. Moreover, inducible knockdown allows the investigation of genes that would be lethal or otherwise poorly tolerated if constitutively knocked down. Lentiviral inducible shRNA vectors are readily available, but unfortunately the process of cloning, screening, and testing shRNAs can be time-consuming and expensive. Therefore, we sought to refine a popular vector (Tet-pLKO-Puro) and streamline the cloning process with efficient protocols so that researchers can more efficiently utilize this powerful tool.

**Methods﻿:**

First, we modified the Tet-pLKO-Puro vector to make it easy (“EZ”) for molecular cloning (EZ-Tet-pLKO-Puro). Our primary modification was to shrink the stuffer region, which allows vector purification via polyethylene glycol precipitation thereby avoiding the need to purify DNA through agarose. In addition, we generated EZ-Tet-pLKO vectors with hygromycin or blasticidin resistance to provide greater flexibility in cell line engineering. Furthermore, we provide a detailed guide for utilizing these vectors, including shRNA design strategy and simplified screening methods.

**Results:**

Notably, we emphasize the importance of loop sequence design and demonstrate that the addition of a single mismatch in the loop stem can greatly improve shRNA efficiency. Lastly, we display the robustness of the system with a doxycycline titration and recovery time course and provide a cost/benefit analysis comparing our system with purchasing pre-designed shRNA vectors.

**Conclusions:**

Our aim was twofold: first, to take a very useful shRNA vector and make it more amenable for molecular cloning and, secondly, to provide a streamlined protocol and rationale for cost-effective design, cloning, and screening of shRNAs. With this knowledge, anyone can take advantage of this powerful tool to inducibly knockdown any gene of their choosing.

## Background

Knockdown of gene expression at the mRNA level via RNA interference (RNAi) is a common method for investigating gene function. For transient knockdown in mammalian cell culture, small interfering RNA (siRNA) is often favored. The benefits of siRNA include commercially available RNA oligos which can be transfected into cells for quick and efficient knockdown. However, siRNA becomes less useful when working with cell types with low transfection efficiency or in experiments that require prolonged gene knockdown [1]. Another common method for utilizing RNAi is short-hairpin RNA (shRNA), synthetic non-coding RNA that utilizes the endogenous microRNA machinery to process functional RNAi. Though not as simple to use as siRNA, shRNA can avoid concerns of low transfection efficiency and temporary knockdown by using retroviral delivery and selection for stable genomic integration [2–4].

Lentiviral shRNA vectors are popular due to their ability to infect nearly any cell type and integrate into the genome of both dividing and non-dividing cells. In 2006, the BROAD institute established the RNAi Consortium to identify and clone multiple shRNA candidate sequences for every gene in the mouse and human genomes [5]. The consortium cloned the shRNA sequences into the pLKO lentiviral vector backbone and has made them available for distribution from GE Healthcare Dharmacon and Sigma-Aldrich. The shRNAs were not all functionally validated but were given a computationally calculated score for predicated efficiency and specificity.

In 2009, Dmitri Wiederschain and colleagues built upon the pLKO vector and made multiple changes, the two most significant of which were the inclusion of the Tet-Repressor gene (TetR) and an H1 promoter containing the TetOperator (TetO) sequence to drive shRNA expression. Together, these modifications allow transcription of shRNA upon the addition of tetracycline, or its analogue doxycycline (Dox), to sequester TetR and relieve repression at the TetO [5, 6]. This vector combines the benefits of lentiviral delivery and inducible gene knockdown, providing many advantages over siRNA or constitutive shRNA. One key advantage is the ability to use the same pool of cells for the controls (no induction) and the test sample (plus inducer), thereby eliminating concerns of transfection/infection efficiency or unintentional clonal selection between ‘empty/non-targeting’ and ‘shRNA’ stable pools. By combining inducible vectors with the list of candidate shRNA sequences from the RNAi consortium it is now possible to induce knockdown of nearly any gene in virtually any cell type.

The Tet-pLKO-Puro vector is a potentially powerful tool, but the process of designing and cloning shRNAs into the vector is not without challenge. In an effort to improve this tool even further we made some modifications to make it more amenable for cloning. Furthermore, we establish clear and efficient protocols for designing and cloning shRNAs into the vector. In addition, we demonstrate the importance of loop design including using a single mismatch to improve shRNA efficiency. With our modified vector (EZ-Tet-pLKO) and a detailed description for designing and cloning shRNAs, we aim to make it easy for anyone to quickly adopt and utilize this tool.

## Results

### Modifications to the Tet-pLKO-Puro vector

**Fig. 1:**
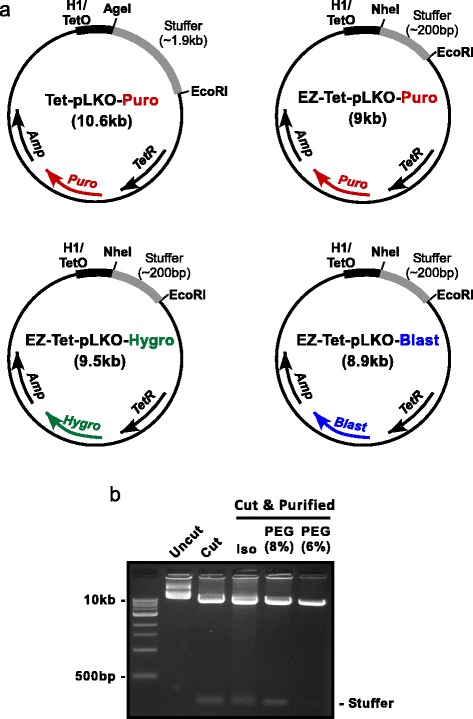
Vector maps and PEG purification. **a** Basic vector maps (not to scale) for the original Tet-pLKO-Puro vector and our modified versions. **b** Agarose gel electrophoresis comparing DNA precipitation methods. 10 μg of EZ-Tet-pLKO vector DNA was co-digested with NheI + EcoRI. The digest was split into three 3 μg aliquots and precipitated with isopropanol (Iso) or polyethylene glycol (PEG) at 6 or 8% concentration. 1 μg of control DNA (uncut and cut) was run alongside 1/3 of the precipitated DNA samples

We started with the Tet-pLKO-Puro vector and modified it to make it more amenable for molecular cloning, terming our version EZ-Tet-pLKO-Puro. First, we used mutagenesis to delete the large non-functional stuffer region (~1.9 kb), leaving a smaller stuffer of ~200 bp (Fig. 1a). Second, we mutated the 5′ AgeI cloning site to an NheI sequence to ameliorate occasional difficulties with inefficient AgeI + EcoRI co-digestion. Additionally, we generated matching vectors with mammalian selection markers for hygromycin (Hygro) or blasticidin (Blast) resistance (Fig. 1a). The smaller stuffer makes it possible to purify cut vector by size-selective DNA precipitation with polyethylene glycol (PEG). To compare precipitation methods, cut DNA was precipitated by isopropanol, 8% PEG, or 6% PEG. The 6% PEG precipitation removed nearly all of the 200 bp stuffer (Fig. 1b). Together, the combination of vector modifications and utilization of PEG precipitation provides a simplified method for preparing cut vector.

### shRNA oligo design

**Fig. 2:**
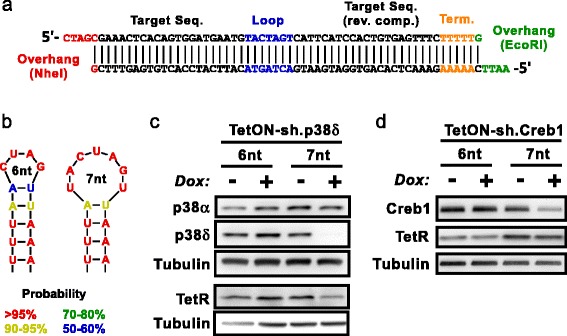
shRNA oligo design and loop comparison. **a** Format for shRNA oligo design. Upper strand is sense oligo, lower strand is anti-sense oligo. **b** Diagram of predicted shRNA loop structure with a basic SpeI sequence (6 nt: ACUAGU) or including a single stem mismatch (7 nt: UACUAGU). Colors correlate to calculated likelihood of the depicted pairing. See methods for details on prediction tool. **c** Immunoblot showing two different pools of iPrEC cells with shRNA against p38δ, with the only difference being a single mismatch in the loop sequence of the shRNA. Cells were treated −/+ Dox for 72 h. TetR was probed on a separate gel. p38α and Tubulin serve as loading controls. **d** Same experiment as **c** using a different pair of shRNAs targeting Creb1. Cells were treated −/+ Dox for 5 days

Developing functional shRNA constructs often requires testing many targeting sequences; therefore, a process for designing shRNAs quickly and efficiently is quite valuable. Targeting sequences were selected as described in the methods section and used to generate sense and antisense shRNA oligos. shRNA oligos contain the following elements: 5′ overhang, targeting sequence, loop, reverse-complement targeting sequence, transcriptional terminator sequence, and 3′ overhang (Fig. 2a). The antisense oligo (bottom strand) is a reverse complement of the sense oligo with complementary overhangs. Without a mismatch, a 6 nt palindrome loop is predicted to collapse to a 4 nt loop and shift the targeting sequence by one base (Fig. 2b). Immortalized prostate epithelial cells (iPrECs) were infected with shRNA lentivirus (sh.p38δ or sh.Creb1) and pools were selected containing the same targeting sequence with or without a single mismatch. Immunoblot showed very efficient knockdown of p38δ with the 7 nt loop and no knockdown with the 6 nt loop (Fig. 2c). Probing for TetR showed that both pools were infected with the lentivirus and had similar expression levels of the lentiviral construct. A similar test was performed using cells containing the sh.Creb1 construct and produced similar results (Fig. 2d). Thus, when designing shRNA sequences it is crucial to consider not only the targeting sequence, but also a mismatch in the loop stem.

### Streamlined colony screening

**Fig. 3:**
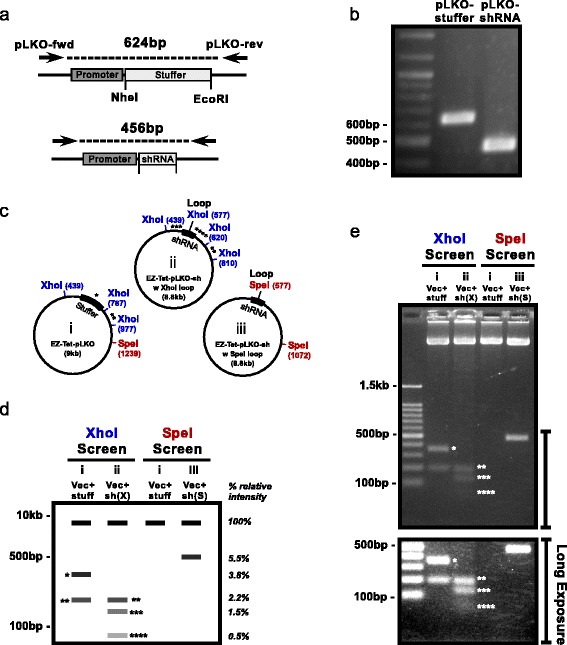
Screening techniques. **a** Diagram showing expected products from PCR screening pLKO ligation-transformed colonies. **b** Agarose gel (2%) with a positive and negative PCR product. **c** Vector maps (not to scale) with XhoI and SpeI restriction digest sites labeled in bp. *Asterisks* indicate corresponding bands in Fig. 3d and e. **d** Diagram showing expected DNA fragments and relative intensity on gel from an XhoI (*blue*) vs SpeI (*red*) shRNA loop restriction digest screen of the plasmids shown in 3c (i - parental EZ- Tet-pLKO vector with stuffer (Vec + stuff), ii - EZ-Tet-pLKO with shRNA XhoI loop (Vec + sh(X)), iii - EZ-Tet-pLKO with shRNA SpeI loop (Vec + sh(S)). (*) is the predicted 348 bp XhoI fragment spanning the stuffer region in the original Tet-pLKO vector (i). In the EZ-Tet-pLKO vector harboring an shRNA with an XhoI site in the loop (ii), XhoI digestion will generate three small fragments, 190 bp (**), 138 bp (***), and 43 bp (****). In the EZ-Tet-pLKO vector harboring an shRNA with an SpeI site in the loop (iii), SpeI digestion will generate a clearly visible diagnostic 500 bp fragment. **e** Agarose gel (2%) with XhoI or SpeI shRNA screens of constructs indicated in 3c (i, ii, iii). Each lane was loaded with 4 μg of digested DNA. Bottom image shows lower part of the same gel with a longer exposure to show the barely detectable 43 bp (****) fragment

After ligation of vector and shRNA oligos the DNA must be transformed into competent bacteria and colonies must be screened. Colony-PCR is a quick way to use small amounts of bacteria directly as template in a PCR reaction. We designed primers to span the stuffer/shRNA insert region, producing a ~450 bp band for positive clones and a ~620 bp band for background vector with retained stuffer (Fig. 3a). PCR product was visualized by agarose gel electrophoresis, which produced clearly identifiable bands for true clones and background colonies (Fig. 3b).

Additionally, clones can be further validated by restriction enzyme (RE) digest screening, which requires a miniprep step to isolate plasmid DNA. The original Tet-pLKO-Puro protocol recommended using an XhoI loop in the hairpin [6, 7]. Because there are already 3 XhoI sites in the parental EZ- Tet-pLKO vector (Fig. 3c-i), introducing a fourth XhoI site in the loop creates four fragments upon digestion (Fig. 3c-ii, d). Furthermore, in the EZ-Tet-pLKO vector two of these bands are so small, 138 bp (***) and 43 bp (****), representing less than 2% of the total DNA (Fig. 3c-ii, d) making it very difficult to visualize on an agarose gel even with a long exposure (Fig. 3e). As a way to simplify and improve the RE screening process, we recommend a SpeI site for loop design (Fig. 3c-iii). When visualized on agarose, a positive SpeI screen produces a clear band at ~500 bp, which is ~5% of total DNA and easily detectable (Fig. 3d, e). We further validated the EZ-Tet-pLKO-shRNA positive clones by Sanger sequencing using the same pLKO-fwd primer as used in the PCR screen. Thus, the combination of colony-PCR as a cheap and quick primary screen and SpeI-based digest as a secondary screen creates a streamlined process for identifying positive shRNA clones.

### Dox Titration and recovery time courses

**Fig. 4:**
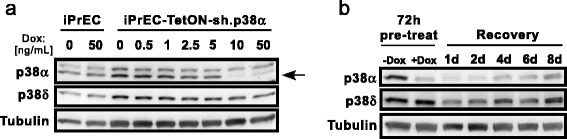
Dox titration and recovery. **a** Immunoblot showing Dox titration with iPrECs containing EZ-Tet-pLKO-sh.p38α. Cells were treated with Dox for 72 h and lysed. Note: the lower band (*arrow pointing*) is p38α. **b** Cells were treated −/+ Dox (50 ng/mL) for 72 h. At that time, two samples were lysed (72 h pre-treated) while another plate of treated cells was split and allowed to recover without Dox for 1–8 days. Note: due to changes in confluency, the ‘pre-treated’ cells have higher basal level of p38 (α and δ) than at day 8

Next, we validated the efficacy of the EZ-Tet-pLKO-Puro vector in cell culture. Cells were infected with lentivirus and pools were selected with puromycin. We performed a titration with Dox (0.5 to 50 ng/mL) and found that as little as 10 ng/mL was sufficient to induce target (p38α) knockdown (Fig. 4a). Furthermore, the target protein can be recovered after removal of Dox. Cells with sh.p38α were treated with Dox for 72 h and then split. Dox was removed and samples were harvested over a recovery time course (Fig. 4b). Recovery of protein began four days after removal of Dox. Thus, the EZ-Tet-pLKO system is both inducible and reversible.

### Cost analysis and comparison

Our method for designing, cloning, screening, and validating lentiviral shRNAs is not only efficient but also cost effective. Most reagents can be found in a standard molecular biology lab (e.g., restriction enzymes, PCR reagents, agarose gel electrophoresis equipment). The EZ-Tet-pLKO plasmids can be acquired from the Addgene repository. The only reagent that is single-use are the shRNA oligos, which are unique for each clone and also the largest single cost. However, once the required reagents are assembled, it only costs ~ $50 in supplies and materials for each new shRNA cloned (Table 1).

**Table 1:** Reagent cost analysis

Reagent	Cost	Source
EZ-Tet-pLKO plasmid	$65	Addgene
PEG-8000	$38 *(per 250 g)*	Sigma
shRNA oligo (sense/antisense)	$22 (*ea.)*	IDT
Screening primer (Fwd/Rev)	$8 *(ea.)*	IDT
Cloning enzymes (PNK, AP, ligase)	$2.50 *(per ligation)*	NEB, Affymetrix
Restriction enzymes	$0.25 *(per digest)*	NEB
PCR reagents	$0.25 *(per reaction)*	Empirical Bioscience
Total cost to clone first shRNA	~$170	
Cost per subsequent shRNA	~$50	

Estimated costs for the various reagents needed in the cloning protocol. Note: Does not include plastic consumables or common lab reagents (e.g., LB media, alcohol, agarose, competent cells) or lentiviral packaging components. Estimated total cost is based on screening 10 colonies per shRNA ligation. The primary cost for subsequent shRNAs are the sense and anti-sense oligos ($44), with the remaining cost coming from consumable enzymes. PNK: Polynucleotide Kinase. AP: Antarctic Phosphatase. IDT: Integrated DNA Technologies. NEB: New England BioLabs.

In addition to the method described here, it is also possible to purchase shRNAs already cloned in lentiviral plasmids from an RNAi Consortium library, such as Sigma Aldrich or Dharmacon. The costs and benefits to using our custom design method vs purchasing vectors (Table 2) out-weigh the others. The primary benefits of our method are the low costs and customizability, the ability to use the improved 7 nt loop, and use of any of three different selection vectors (Puro, Hygro, or Blast). The alternative options are to purchase shRNAs already cloned into a vector at a cost of $50–$100 (non-inducible) or $400–$450 (Dox inducible), and they are limited to the 6 nt loop. In addition, lentiviral particles can also be purchased which allows for immediate infection but come at a high cost (> $1,000). Though the commercial options may be quicker, the cost, customizability, versatility, inducibility, and more efficient 7 nt loop of the EZ-Tet-pLKO method makes it a better option overall.

**Table 2:** Cost/benefit comparison of lentiviral shRNA methods

Method	Cost	Unit	Benefits	Drawbacks
EZ-Tet-pLKO	$50	per shRNA	Low cost, Customizable, Puro/Hygro/Blast Efficient 7 nt loop	2–3 day process
TRC library (non-inducible)	$208/$330 *(glycerol stock)*	per shRNA/Set of 3–6	No cloning required	Non-inducible, 6 nt loop
TRC library (Dox-inducible)	$450/$1100 *(glycerol stock)*	per shRNA/ Set of 3	No cloning required	High cost, 6 nt loop
	$1195 *(viral particles)*	1 unique or 3 mixed	Ready to use	

Comparison of cost/benefits of our cloning method versus other sources of TRC (The RNAi Consortium) library shRNAs. Note: Costs here are based on Dharmacon prices (http://dharmacon.gelifesciences.com/applications/rna-interference/shrna/, last accessed Dec. 6^th^, 2016).

### Efficiency

The large stuffer (1.9 kb) region between the cloning sites in the original Tet-pLKO-Puro vector necessitated the tedious process of gel purifying the vector fragment. By reducing the stuffer to 200 bp we were able to more efficiently and quickly isolate the purified cut vector with PEG. Use of the original vector with the long stuffer resulted in the same number of colonies in the vector-only ligated plate as on the insert ligation plate, necessitating excessive screening of >50 colonies to find 1 transformant with the insert (i.e., 2%). With the shorter stuffer, there were typically ~0–20 colonies on the vector-only ligation plate and >20–100 colonies on the insert ligation plate, equating to an efficiency of ~10–50 colonies per ng of cut vector (# colonies × 1000/100 (LB dilution) × 20/4 (ligation reaction dilution) × 1/100 (ng vector DNA per μL ligation reaction). Furthermore, ~80% of these colonies had the desired insert. In addition, the shorter stuffer allows for improved PCR screening and saves at least a day by not having to wait to grow up the colonies before standard restriction enzyme screening.

## Discussion

The EZ-Tet-pLKO vector together with our detailed methods provides a descriptive guide to efficiently utilize inducible shRNAs. Though we have focused on a modified pLKO vector, the principles of shRNA design and screening could be applied to many other cloning scenarios. Our primary modification to the vector was to shrink the stuffer region. The stuffer is non-functional DNA, and we chose to keep a small 200 bp region so that double-cut vector could clearly be visualized separately from linearized single-cut vector on agarose. Moreover, retaining a small stuffer allows for size-selective precipitation of cut vector via PEG [8]. Compared to alcohol precipitation and gel extraction, PEG precipitation is faster, provides cleaner DNA, and avoids concerns of potential DNA damage from UV exposure [9, 10]. We also sought to emphasize the importance of using a proper loop design for shRNAs including adding a stem mismatch [11]. The inclusion of a mismatch in the loop region can aid hairpin formation by preventing loop collapse and thus shifting the targeting sequence, which can disrupt proper DICER binding and target mRNA cleavage [12, 13]. The mismatch was not always necessary for proper shRNA function (not shown), but in at least the two cases reported here it was crucial and should always be included to maximize the chances of developing a successful shRNA construct.

Though we sought to make our protocol as easy as possible, there are some potential areas of difficulty that may be avoided by taking extra precautions. One critical detail is that the DNA pellet precipitated by PEG can often be invisible, so extra caution should be taken when decanting the supernatant after centrifugation. If recovery is consistency low, consider trying 7 and 8% PEG precipitations to increase precipitation efficiency at the tradeoff for slightly more stuffer retention. When transforming the ligation into bacteria, it is important to use recombination-deficient *E.coli* strains (such as NEB-Stable) in order to minimize unwanted recombinations due to lentiviral LTR sequences. When sequencing clones, be aware that shRNA hairpin sequences can sometimes cause early termination when read by Sanger sequencing and may (but not always) require the use of specialized sequencing protocols for dealing with RNAi constructs [14, 15]. Lastly, freshly prepared lentivirus is preferred when infecting cells, though frozen virus can be used with ~50% decrease in infectivity for each freeze-thaw cycle.

One important caveat with the Dox-inducible system is that at high doses (>1 μg/mL), Dox can have detrimental effects on cell viability via disruption of mitochondrial function [16]. In our experience, we observed viability effects from prolonged treatment (>4 days) at 500 ng/mL but saw no effects from a 2-week treatment at 50 ng/mL (not shown). As an extra control, the parent cell line (without lentiviral infection) can be treated with Dox to check specifically for effects on cell viability. In most cases a 10–50 ng/mL dose of Dox should be well tolerated but that should be tested by the end user in their particular cell line as a precaution.

The timing of gene knockdown and recovery is not universal. For most genes 72 h is sufficient to see knockdown at the protein level. However, this is highly dependent on protein stability. Longer-lived proteins (e.g., membrane-bound receptors, housekeeping proteins) may take up to a week for proper knockdown. We observed p38α knockdown at 72 h, but Creb1 knockdown was not observed until at least day five of Dox induction. Likewise, protein recovery will be highly dependent on the transcription rate of the gene so that lower expressed genes will take longer to recover. Furthermore, cell confluency and proliferation rate will also affect the rate of protein synthesis and turnover, thus affecting Dox knockdown and recovery timing. All these factors need to be considered when designing temporally-sensitive experiments and will be cell and context specific.

When testing new shRNA constructs, transduced cells lines need to be validated. Knockdown at the mRNA level can usually be seen by qRT-PCR at 24–48 h. However, as previously mentioned, protein knockdown can take up to five days or longer potentially. A good control to include when testing new pools is to probe an immunoblot for the TetR protein to confirm that the selected pool of cells has robust expression of the lentiviral vector. Likewise, if comparing pools or clones, those with highest TetR expression often show the greatest knockdown (not shown). When targeting a new gene, we recommend starting with at least three different targeting sequences with the expectation that one or two will work efficiently.

Lastly, we also sought to aid researchers by designing Hygro and Blast resistant variants of the EZ-Tet-pLKO vector, thus providing more flexibility in creating multiple genetic engineered cell lines. By combining all three vectors in one cell line it would be possible to knockdown two or three targets simultaneously upon Dox treatment. In addition to the Tet inducible system, there are other inducible shRNA vectors that can prove useful and are commercially available, such as cumate or IPTG-inducible vectors [17, 18]. With some creativity and strategy it would also be possible to create cells with multiple shRNAs, each activated by different inducers. Moreover, inducible shRNAs could be combined with inducible cDNA expression systems to test overexpression and knockdown simultaneously or sequentially [19]. Use of inducible vectors with various selection markers opens the door for greater quantity and variety of questions that can be addressed with molecular biology.

## Conclusions

Inducible shRNAs are a very powerful tool when used properly. We sought to provide a guide to allow more people to more easily use this system with our EZ-Tet-pLKO vector. There are lots of ways to manipulate gene expression, including the recent advent of CRISPR/Cas9 technologies. Though the potential of CRISPR is great, it is not without serious limitations, including inability to study genes with lethal knockdown phenotypes and the reliance on selecting clonal populations for cell culture studies [20]. In addition to the cell culture uses shown here, the pLKO system is also useful in vivo, for example with tumor xenografts which can be induced to knockdown a gene upon addition of Dox to the animal food or water [21]. Our goal with this report was to take the already proven Tet-pLKO-Puro system and refine it further. With these new EZ-Tet-pLKO vectors and protocols, researchers will find this tool to be more versatile and user-friendly than ever.

## Methods

### pLKO vector modifications

The Tet-pLKO-Puro plasmid was ordered from Addgene (Plasmid 21915) [6, 7]. Mutagenesis was performed using the QuikChange II Site Directed Mutagenesis kit (Aligent). Bases 222–1869 of the stuffer region between the AgeI and EcoRI cloning sites were deleted. The deletion was performed by inserting an EcoRI site at base 222 of the stuffer (primer 5′- GCTACTCCACCACTTGAATTCCTAAGCGGTCAGC). The vector was then digested with EcoRI, re-ligated, and clones were screened for those that ligated the new EcoRI site directly to the 3′ cloning site, thus excising the bulk of the stuffer region and preserving the 3′ cloning site. Mutagenesis was then used to mutate the AgeI restriction site to an NheI sequence (primer 5′- TATCAGTGATAGAGACGCTAGCGTGTTGTAAATGAGCA). The EZ-Tet-pLKO-Hygro vector was made by PCR subcloning the Hygro resistance gene from the pGL4.15 vector (Promega) using the following primers: 5′-ATTATGGATCCATGAAGAAGCCCGAACTC and 5′- ATTATGACGTCTTAAACTCGACCTACCTC. The EZ-Tet-pLKO-Blast construct was made by PCR subcloning the Blast resistance gene from pLenti-CMV-rtTA3-Blast (Addgene 26429). For PCR cloning, inserts were amplified with Q5 high fidelity polymerase (NEB) and ligated into Tet-pLKO-Puro between the BamHI and AatII RE sites. All experiments were carried out with the Puro variant.

### Vector digest and PEG precipitation

Vector can be prepared by co-digesting EZ-Tet-pLKO-Puro DNA with NheI and EcoRI (NEB). A typical digest consisted of 5 μg of vector DNA with 20 u of each enzyme in a 50 μL digest volume for at least 3 h at 37 °C. Cut vector was then dephosphorylated with Antarctic Phosphatase (NEB) using the manufacturer’s protocol and supplementing the 50 μL digest reaction with AP buffer, enzyme, and water to make a 60 μL reaction volume. Cut vector was then diluted with water to a 200 μL volume in a 1.5 mL Eppendorf tube. PEG was used to precipitate the DNA and exclude the 200 bp excised stuffer. We first prepared 2× stock of 12% (w/v) PEG-8000 and 20 mM Magnesium Chloride. The 2× stock was then added 1:1 to the cut and dephosphorylated DNA sample. The DNA/PEG mixture was gently mixed by inverting the tube a few times and left to sit at room temperature for at least 1 h. After the incubation, the DNA was centrifuged at 15,000 RCF in a bench top centrifuge (Eppendorf 5415D) for 40 min. The length of the incubation and spin are critical; any less time can greatly decrease recovery. Next, 500 μL of 70% ethanol was added to wash the DNA pellet, which was then spun again for 5–10 min. The ethanol was then aspirated and the wash was repeated once more. After the second wash the DNA pellet was allowed to air dry and then suspended in water (typically ~50 μL). DNA was then quantified by Qubit (Q32850, ThermoFisher). Accurate quantification is important for successful cloning. Typically DNA recovery following 6% PEG precipitation is ~50%.

### shRNA oligo design and loop prediction

shRNA targeting sequences were chosen from the BROAD RNAi Consortium database (http://www.broadinstitute.org/rnai/trc). shRNA targeting sequences (with RNAi consortium ID) are as follows: p38α (TRCN0000196472), p38δ (TRCN0000197043), Creb1 (TRCN0000226466). Oligos were designed as described in Fig. 2 and ordered from Integrated DNA Technologies. The RNA folding probability values in Fig. 2 were calculated using RNAstructure software (v5.7) by Reuter et al. [22] (http://rna.urmc.rochester.edu/RNAstructure.html).

### shRNA oligo preparation

Sense and antisense shRNA oligos were suspended at 100 μM in duplex buffer (100 mM Potassium Acetate, 30 mM HEPES, pH 7.5). Next, 20 μL (2 μ-mol) of each oligo were combined and annealed using a thermalcycler (Labnet TC9600-G) with a program set to start at 95 °C and drop ~5°/min down to room temperature. Alternately, DNA can be annealed by placing in a beaker of boiling water and moved off the heater to cool slowly to room temperature. The annealed oligos were then diluted with water to 360 μL total and precipitated with ethanol (added 40 μL of 3 M sodium acetate and 1 mL ethanol). DNA was centrifuged for 30 min at 15,000 RCF in a bench top centrifuge, washed twice with 70% ethanol, and suspended in 500 μL water. Annealed oligo DNA was then quantified by Qubit (Q32850, ThermoFisher). Synthesized oligos do not contain phosphorylated overhangs, so annealed oligo was treated with T4 poly-nucleotide kinase (M0201, NEB) and heat inactivated, according to the manufacturer’s protocol.

### Ligation and transformation

Prepared vector (cut, dephosphorylated, and PEG purified) was diluted to a working concentration of ~20–100 ng/μL if needed. Phosphorylated oligos were diluted (from the heat-inactivated PNK reaction) to a 1 ng/μL working concentration. Ligations were performed using the LigateIT rapid ligase kit (78400, Affymetrix) with 100 ng vector DNA and an 8:1 insert:vector molar ratio. A vector-only ligation was also prepared to control for incompletely digested and/or re-ligated vector derived colonies. Next, 2 μL of the ligation reactions were transformed into Stbl3 (Life Technologies) or NEB-Stable (NEB) chemically competent *E. coli*. Competent cells were incubated on ice for 30 min with 4 μL of ligation DNA, then heat shocked at 42 °C for 40 s and returned to ice for 1 min. Then, 1 mL of LB media was then added to the cells and they were allowed to recover at 37 °C for 30 min, after which time 100–200 μL was plated on LB-agar plates containing 100 μg/mL ampicillin and incubated 12–16 h at 37 °C.

### PCR screen

Colony-PCR was used to screen bacteria for successfully ligated clones. Primers used were as follows: pLKO-Fwd 5′- ATTAGTGAACGGATCTCGACGG; pLKO-rev 5′- AACCCAGGGCTGCCTTGG. To set up the PCR reactions, first 15 μL of water was added to PCR tubes. Colony inoculation was performed by touching a p10 pipette tip to a colony, then mixing it in the desired PCR tube with the water, and then dotting ~1 uL on a labeled fresh LB agar (+amp) plate to keep track of the colony. A positive control is always included by adding ~ 1–10 ng of EZ-Tet-pLKO-Puro plasmid to 15 μL water. PCR was performed using Emprical Bioscience Taq and buffer (TP-MG-500). A master mix was made containing (per reaction): 2.5 μL of 10× Taq Buffer, 0.2 μL of Taq enzyme, 2 μL of 25 mM magnesium chloride, 0.2 μL of each primer (fwd and rev, 100 μM stocks), and 3.9 μL water. Then, 10 μL of the master mix was then added to the 15 μL of inoculated water which served as the template. Thermalcycler settings used were as follows: 1× [95 °C for 2 min], 35× [95° for 30 s, 68 °C for 45 s], 1× [72 °C for 1 min]. DNA was then run on 2% agarose for visualization with a DNA ladder (N3231 or N3232, NEB). Positive clones can then be further validated by RE screening or sent directly for Sanger sequencing using the pLKO-Fwd primer.

### Restriction enzyme digest screen

Clones were minipreped by alkaline lysis. DNA was digested using the SpeI restriction enzyme (NEB). A standard reaction condition was ~3 μg of DNA digested with 10 u of enzyme in a 50 μL reaction for at least 1 h at 37 °C. Digest reaction (10–20 μL) was then run out on a 2% agarose gel.

### Cell culture

iPrEC cells were grown in KSFM with included supplements (17005042, Gibco) and 30 u/mL Pen/Strep (Gibco). For shRNA induction 50–100 ng/mL Dox (Sigma) was used. HEK293FT cells were used for lentivirus production and maintained in DMEM (11995, Gibco) with 2 mM L-glutamine, 10% fetal bovine serum (FBS), and 30 u/mL Pen/Strep. During transfection and infection, cells were grown without antibiotics and for infection were grown with heat inactivated serum (30 min at 56 °C) to avoid immune complement interference. Cells were maintained at 37 °C with 5% CO_2_.

### Virus production/infection

pLKO constructs were used to make lentivirus in HEK293FT cells using the ViraPower system (K497500, Invitrogen). One T75 flask was needed per viral construct, which were first coated with 2 μg/mL PolyD lysine in PBS for 1 h at 37 °C and then seeded with 5 million cells and left overnight at 37 °C. The next day, cells were switched to antibiotic-free media with heat-inactivated serum and transfected (Lipofectamine2000, ThermoFisher) with packaging plasmids (5 μg each: pLP1, pLP2, pVSV-G) and the desired pLKO construct or a GFP lentiviral vector as control. At 24 h post-transfection, media was changed to the target cell media (without antibiotics). HEK293FT cells were then returned to 37 °C for 48 h to produce viral particles. Viral media was collected in 15 mL conical tubes and centrifuged for 10 min at 1500 RPM in a swinging bucket centrifuge (Megafuge 1.0R) to pellet cell debris. Next, the viral media was filtered by syringe through a 0.45 μM, low protein binding filter (28145–505, VWR). Cells were typically infected by first adding half the volume with normal growth media (no antibiotics, heat inactivated serum) and half volume with the filtered viral media plus polybrene to a 5 μg/mL final concentration to improve infection rate. Infected cells were incubated 48–72 h and then given fresh growth media for 24–48 h before beginning selection. A lentivirus containing GFP was used as a positive control for viral production/infection and to estimate the percentage of infected cells. GFP was detected by fluorescence microscopy 48–72 h after infection.

### Immunoblot

Cells were lysed in MAPK lysis buffer (50 mM Tris, pH 7.5, 0.5 mM EDTA, 50 mM NaF, 100 mM NaCl, 50 mM β-glycerol phosphate, 5 mM Sodium Pyrophosphate, 1% TritonX100) or RIPA lysis buffer (10 mM Tris, pH 7.5, 1 mM EDTA, 158 mM NaCl, 0.1% SDS, 1% Sodium Deoxycholate, 1% TritonX100). Cells were chilled, washed, and then lysis buffer was added and plates sat for 30 min on ice. Cells were then scrapped, centrifuged, and protein was quantified by BCA assay (Pierce). Equivalent amounts of 30–50 μg of denatured protein per sample was run on Novex SDS polyacrylamide tris-glycine gels (Life Technologies). Protein was then transferred onto PVDF membrane and blocked in 5% BSA/TBST for 1 h at room temp. Primary and secondary antibodies were diluted in blocking buffer. Primary antibodies were probed either 2–3 h at room temp or overnight at 4 °C while all secondary antibodies were probed 1 h at room temp. Luminol chemiluminescence was used with a Bio-Rad Chemi-Doc imaging system with CCD camera to image blots and analyzed on Quantity One software v4.5.2. The following antibodies were used: p38α at 1:2000 (CST 9218), p38δ at 1:1000 (Santa Cruz sc-136063), Tubulin at 1:10,000 (Sigma T9026), Creb1 at 1:1000 (CST 4820), and TetR at 1:2000 (Clone Tech 631131).

## Abbreviations

Dox: Doxycycline
FBS: Fetal bovine serum
iPrEC: Immortalized prostate epithelial cell
PEG: Polyethylene glycol
RE: Restriction enzyme
Tet: Tetracycline
TetO: Tet operator
TetR: Tet Repressor
TRC: The RNAi Consortium
